# Cognitive, behavioral, and brain functional connectivity correlates of fatigue in amyotrophic lateral sclerosis

**DOI:** 10.1002/brb3.2931

**Published:** 2023-06-22

**Authors:** Francesca Trojsi, Federica Di Nardo, Giulia D'Alvano, Carla Passaniti, Minoo Sharbafshaaer, Fabrizio Canale, Antonio Russo, Marcello Silvestro, Luigi Lavorgna, Mario Cirillo, Fabrizio Esposito, Gioacchino Tedeschi, Mattia Siciliano

**Affiliations:** ^1^ Department of Advanced Medical and Surgical Sciences, MRI Research Center Università degli Studi della Campania “Luigi Vanvitelli” Naples Italy; ^2^ First Division of Neurology University Hospital Università degli studi della Campania “Luigi Vanvitelli” Naples Italy

**Keywords:** amyotrophic lateral sclerosis, fatigue, functional connectivity, resting‐state functional MRI

## Abstract

**Introduction:**

Fatigue is defined as a symptom of exhaustion unexplained by drug effects or psychiatric disorders and comprises two main components (i.e., central or “mental” and peripheral or “physical” components), both influencing global disability in amyotrophic lateral sclerosis (ALS). We aim at investigating the clinical correlations between “physical” and “mental” components of fatigue, measured by the Multidimensional Fatigue Inventory scale, and motor and cognitive/behavioral disability in a large sample of patients with ALS. We also investigated the correlations between these measures of fatigue and resting‐state functional connectivity of brain functional magnetic resonance imaging (RS‐fMRI) large‐scale networks in a subset of patients.

**Methods:**

One hundred and thirty ALS patients were assessed for motor disability, cognitive and behavioral dysfunctions, fatigue, anxiety, apathy, and daytime sleepiness. Moreover, the collected clinical parameters were correlated with RS‐fMRI functional connectivity changes in the large‐scale brain networks of 30 ALS patients who underwent MRI.

**Results:**

Multivariate correlation analysis revealed that “physical” fatigue was related to anxiety and respiratory dysfunction, while “mental” fatigue was related to memory impairment and apathy. Moreover, the mental fatigue score was directly related to functional connectivity in the right and left insula (within the salience network), and inversely related to functional connectivity in the left middle temporal gyrus (within the default mode network).

**Conclusions:**

Although the “physical” component of fatigue may be influenced by the disease itself, in ALS the “mental” component of fatigue correlates with cognitive and behavioral impairment, as well as with alterations of functional connectivity in extra‐motor networks.

## Graphical abstract

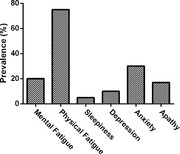

## 1 INTRODUCTION

Fatigue is a typical symptom associated with neurological diseases, described as an overwhelming sense of tiredness, lack of energy, and feeling of exhaustion (Krupp & Pollina, 1996). Fatigue is also reported in amyotrophic lateral sclerosis (ALS) as “a reversible motor weakness and whole‐body tiredness that is predominantly brought on by muscular exertion and is partially relieved by rest” (Gibbons et al., 2013a). In ALS, fatigue can be experienced predominantly as general (feelings of whole‐body tiredness) and physical (reversible motor weakness) (Gibbons et al., 2011; Lou et al., 2003). The former type of fatigue is a pervasive feeling of tiredness or lethargy, different from sleepiness, which is attributed to central mechanisms and is also known as “mental” fatigue. Conversely, the second type of fatigue consists of a decline in the ability of a muscle to contract to maximum force, also known as “physical” fatigue.

Several studies have found that fatigue has a negative impact on the quality of life of ALS patients due to its association with psychological distress, social withdrawal, and respiratory dysfunction with dyspnea (Gibbons et al., 2013b; Young et al., 2022). Importantly, a recent systematic review and meta‐analysis has shown that exercise training could not improve the fatigue severity in ALS patients (Rahmati & Malakoutinia, 2021). However, although it plays a crucial role in influencing global disability in ALS, the cognitive and behavioral correlates of fatigue as well as brain functional connectivity changes associated with this dichotomic construct have not been elucidated. Based on these considerations, to fill in these gaps, we performed a neuropsychological and magnetic resonance imaging (MRI) cross‐sectional study in a cohort of patients with newly diagnosed ALS who underwent assessment of cognitive and behavioral functions, fatigue, anxiety, apathy, and daytime sleepiness, and a brain functional MRI (fMRI) examination exploring resting‐state fMRI (RS‐fMRI) connectivity patterns of brain damage as soon as possible in their disease course after diagnosis. We expected to clarify the cognitive, behavioral, and brain RS‐fMRI correlates of both components of fatigue in the studied cohort of ALS patients.

## 2 METHODS

### 2.1 Case selection

One hundred and thirty patients (80 males; mean age 60.39 ± 9.38) with definite, clinical, or laboratory‐supported probable ALS, according to El‐Escorial revised criteria (Brooks et al., 2000), were consecutively recruited at the First Division of Neurology of the University of Campania “Luigi Vanvitelli” (Naples, Italy) from June 2020 to January 2022. Patients were required to meet the following criteria: showing classical, bulbar, flail limbs, or pyramidal phenotypes (Chiò et al., 2011); disease onset not earlier than 18 months from enrollment; the age of onset of 40 years or older.

As for the clinical assessment, the disability status was measured by the ALS Functional Rating Scale—Revised (ALSFRS‐R) total score (0–48, with the lower total reflecting higher disability) and subscores (i.e., bulbar, fine motor, gross motor, and respiratory subscores) (Cedarbaum et al., 1999), and the upper motor neuron (UMN) score, an index of pyramidal dysfunction through the evaluation of the number of pathologic reflexes elicited from 15 body sites (Turner et al., 2004). Disease duration was calculated from symptom onset to the scan date in months.

To assess cognitive and behavioral profiles, all patients underwent the Italian version of the Edinburgh Cognitive and Behavioral ALS Screen (ECAS) (Poletti et al., 2016; Siciliano et al., 2017), collecting total scores with its subscores (i.e., executive functions, fluency, language, memory, and visuospatial abilities). Lower scores indicate worse cognitive performance. An age‐ and‐education‐adjusted cut‐off score of 67.06 points was used for assessing cognitive decline (Siciliano et al., 2017).

Depressive and anxious symptoms were measured by Beck Depression Inventory‐II (BDI‐II) (Beck et al., 1996), and State‐Trait Anxiety Inventory (STAI) Form Y‐1 (Spielberger, 1983). Cut‐off scores of 20 points for BDI‐II (Beck et al., 1996) and 41 points for STAI form Y‐1 (Siciliano et al., 2019) were adopted for revealing the presence of clinically significant depressive and anxious symptoms, respectively.

Apathetic symptoms were assessed by using Dimensional Apathy Scale (DAS) (Radakovic & Abrahams, 2014; Santangelo et al., 2017) total score with its subscales (i.e., executive, emotional, cognitive, and behavioral initiation). A cut‐off score of 27 points was employed for identifying clinically significant apathetic symptoms (Santangelo et al., 2017). Excessive daytime sleepiness was rated by Epworth Sleepiness Scale (ESS) (Johns, 1991). A cut‐off score of 10 points was used for ascertaining the presence of clinically significant excessive daytime somnolence (Johns., 1991). Fatigue was measured by the Multidimensional Fatigue Inventory (Smets et al., 1995) total score with its subscales (i.e., physical fatigue, mental fatigue, general fatigue, reduced activity, and reduced motivation). A uniform cut‐off score of 13 points was adopted for revealing the presence of mental and physical fatigue. This was in accordance with previously published studies in neurological populations (e.g., Parkinson's disease (Skorvanek et al., 2015)) as well as in chronic fatigue syndrome (Reeves et al., 2005). For all tests described above, except for ALSFRS‐R and ECAS, higher scores indicate more marked symptoms.

Genetic analysis was performed in all patients, exploring *C9orf72* repeat expansion and mutations of *SOD1*, *TARDBP*, and *FUS/TLS*. No mutations of these genes were reported.

Twenty‐seven healthy control subjects (HC) (21 males; mean age 58.48 ± 12.07) were enrolled by “word of mouth” and among caregivers’ friends. They were age‐, sex‐, and education‐matched with the enrolled ALS patients and unrelated to them. Moreover, they had no comorbid neurological, psychiatric, or medical conditions. They underwent mini mental state examination (MMSE) and their scores were ≥28.

For all subjects, exclusion criteria were medical illnesses or substance abuse that could interfere with cognitive functioning; any other major systemic, psychiatric, or neurological diseases, including dementia; other causes of brain damage, including lacunae and extensive cerebrovascular disorders at MRI (e.g., regarding the enrolled ALS patients, at least one MRI exam was acquired at diagnosis); and a vital capacity lower than 70% of the predicted value.

The research was conducted according to the principles expressed in the Declaration of Helsinki. Ethics approval was obtained from the Ethics Committee of the University of Campania “Luigi Vanvitelli” (n. 591/2018). Patient/HC or family has written informed consent was obtained from each participant.

### 2.2 Statistical analysis: between‐groups comparisons of clinical and neuropsychological data

All data were tested for normality, and values between –1 and +1 for asymmetry and kurtosis were considered acceptable (Hays et al., 1993). According to the appropriate cut‐off scores, we estimated the prevalence of extra‐motor features in our sample. Moreover, we used bivariate regression analyses to identify demographic, clinical, motor, and extra‐motor variables associated with the severity of mental and physical fatigue. Then, in the next analytic step, two multivariate regression analyses (forced entry method), one for mental fatigue and one for physical fatigue, were performed, entering variables that resulted in being statistically significant in the first step (*p* < .05), using subscale scores rather than total scores, when applicable. We used the Statistical Package for Social Science (SPSS, v25) for all analyses, with *p* values < .05 considered statistically significant.

### 2.3 MRI analysis

#### 2.3.1 Magnetic resonance imaging

ALS patients and HC who gave their consent underwent an MRI exam on a 3T scanner equipped with an 8‐channel parallel head coil (General Electric Healthcare, Milwaukee, Wisconsin). The imaging protocol included three‐dimensional T1‐weighted sagittal images (gradient‐echo sequence Inversion Recovery prepared Fast Spoiled Gradient Recalled‐echo, repetition time = 6.988 ms, inversion time = 650 ms, echo time = 3.0 ms, flip angle = 9, voxel size = 1 × 1 × 1 mm^3^; acquisition time = about 7 min); RS‐fMRI sequence, consisting of 320 volumes of a repeated gradient‐echo echo‐planar imaging T2*‐weighted sequence (repetition time = 1500 ms, echo time = 19 ms, nr. of axial slices = 44, matrix = 96 × 96, field of view = 288 mm, thickness = 3 mm, interslice gap = 0 mm, voxel size 3 × 3 × 3 mm3; acquisition time = about 8 min); T2‐fluid attenuation inversion recovery to exclude severe cerebrovascular disease according to standard clinical neuroradiological criteria on visual inspection by three experienced radiologists. During the functional scan, subjects were asked to simply stay motionless, awake, and relax and to keep their eyes closed. No visual or auditory stimuli were presented at any time during functional scanning. The total duration of each scan was about 38 min.

#### 2.3.2 RS‐fMRI data preparation and preprocessing

Standard functional image data preparation, preprocessing, statistical analysis, and visualization were performed with the software BrainVoyager QX (Brain Innovation BV, Maastricht, The Netherlands). Data preprocessing included the correction for slice scan timing acquisition, a three‐dimensional rigid‐body motion correction based on a 6‐parameter rigid‐body alignment to correct for minor head movements, and the application of a temporal high‐pass filter with cut‐off set to 0.008 Hz and a spatial smoothing of image series with a 6 mm full‐width at half‐maximum isotropic Gaussian kernel 3 cycles per time course. Translational motion parameters were verified to be always less than 1 functional voxel for all included participants. The mean frame‐wise displacement (FD) (i.e., a surrogate metric of head motion accounting for intravoxel residual motion effects) was also estimated from the translational and rotational parameters and a typical cut‐off of 0.5 mm was applied (Power et al., 2014). We further verified that there were no statistically significant differences in the mean FD when carrying group comparisons. Structural and functional data were coregistered and spatially normalized to the Talairach standard space using a 12‐parameter affine transformation.

#### 2.3.3 Resting‐State Network (RSN) functional connectivity analysis

To extract RSN maps, single‐subject and group‐level independent component analyses (ICA) were carried out on the preprocessed functional time series using 2 plug‐in extensions of BrainVoyager QX (Goebel et al., 2006), respectively, implementing the fastICA algorithm (Hyvärinen et al., 2006) and the self‐organizing group ICA algorithm (Esposito et al., 2005). Furthermore, the ICASSO procedure (Himberg et al., 2004) was applied to the extraction of individual ICA components according to previously described methods (Trojsi et al., 2021).

For every single subject, 50 independent components were extracted (corresponding to 1/6th of the number of time points) (Greicius et al., 2007) and scaled to spatial *z* scores (i.e., the number of standard deviations of their whole‐brain spatial distribution). To generate group components and allow for population‐level inferences in each RSN, all individual component maps from all subjects were “clustered” in the subject space according to the mutual similarities of their whole‐brain distributions using the self‐organizing group ICA algorithm. Thereby, all 50 individual independent components were uniquely assigned to 1 out of 50 “clusters” of independent components. Once the components belonging to a cluster were retrieved, the corresponding maps were averaged, and the resulting map was assumed to the representative of the cluster. The 50 single‐group average maps were visually inspected to recognize the spatial patterns associated with the main RSNs (Smith et al., 2009). For this purpose, single‐group 1‐sample *t* tests were used to analyze the whole‐brain distribution of the components in each group separately and the resulting t maps were thresholded at *p* = .05 (Bonferroni corrected over the entire brain) after regressing out age and gender from the series of individual maps at each voxel. An inclusive mask was finally created from the healthy control group maps and used to define the search volume for within‐network two‐group comparisons. These comparisons were performed by fitting a one‐way analysis of variance (one‐way ANOVA) model that included one between‐subject factor with two levels: healthy controls and ALS patients and then calculating post hoc *t* contrasts for obtaining between‐group *t* maps. To correct the resulting *t* maps for multiple comparisons, regional effects within the search volume were only considered significant for compact clusters emerging from the joint application of a voxel‐ and a cluster‐level threshold. The cluster‐level threshold was estimated nonparametrically with a randomization approach: we calculated the FWHM from each RSN t map for the healthy control group and then, starting from an initial (uncorrected) threshold of *p* = .001 (or *p* = .005) applied to all voxels, a minimum cluster size was calculated that protected against false‐positive clusters at 5% after 1000 Monte–Carlo simulations (Forman et al., 1995). Individual *z* scores from regions identified in the above analysis were also extracted and used in linear correlation analysis in the ALS patients’ group with MFI scores performed by the “corrcoef” function of MATLAB. For these regional analyses, we used the Pearson's linear correlation coefficient and a statistical significance level of *p* < .05 (Bonferroni corrected).

## 3 RESULTS

### 3.1 Demographics and neuropsychological variables

**FIGURE 1:**
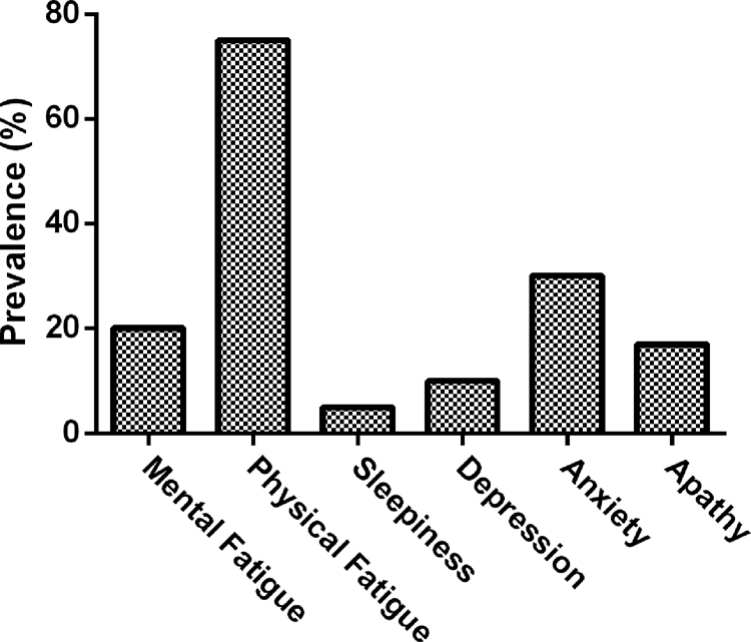
Prevalence of extra‐motor symptoms in the enrolled patients.

We enrolled 130 patients and 27 healthy controls, whose descriptive statistics are reported in Table 1. Asymmetry and kurtosis were acceptable for all continuous variables. The prevalence of extra‐motor features is shown in Figure 1.

**TABLE 1:** Descriptive statics of ALS patients and healthy controls; data are shown as mean (standard deviation) or count (percentage)

Variable	ALS (*n* = 130)	HC (*n* = 27)	*F* test/χ^2^	*p* Value
*Demographics*				
Age, years	60.39 (9.38)	58.48 (12.07)	0.83	.362
Education, years	10.52 (4.47)	10.63 (3.87)	0.01	.909
Sex, male	80 (61.50%)	21 (77.80%)	2.56	.109
*Clinical and motor features*				
Disease Duration, months	27.19 (31.35)	–	–	–
ALSFRS‐R total score	37.09 (6.98)	–	–	–
*ALSFRS‐R subscores*				
Bulbar	10.54 (1.96)	–	–	–
Fine motor	8.12 (3.23)	–	–	–
Gross motor	7.30 (3.19)	–	–	–
Respiratory	11.10 (1.78)	–	–	–
Upper motor neuron score	8.31 (4.28)	–	–	–
Patients under NIV	10 (7.69%)	–	–	–
Patients with PEG	3 (2.30%)	–	–	–
Patients using Riluzole	119 (91.53%)	–	–	–
*Extra‐motor features*				
ECAS total score	86.45 (22.88)	–	–	–
*ECAS subscores*				
Executive functions	26.54 (9.98)	–	–	–
Fluency	15.68 (6.68)	–	–	–
Language	20.73 (5.06)	–	–	–
Memory	12.75 (5.21)	–	–	–
Visuospatial abilities	10.69 (1.49)	–	–	–
Beck Depression Inventory‐II	9.63 (8.37)	–	–	–
STAI form Y‐1	37.49 (11.50)	–	–	–
DAS total score	19.70 (9.20)	–	–	–
*DAS subscales*				
Executive	3.64 (3.86)	–	–	–
Emotional	6.78 (3.81)	–	–	–
Cognitive and behavioral initiation	9.33 (5.60)	–	–	–
Epworth Sleepiness Scale	4.60 (3.22)	–	–	–
*Fatigue measures*				
MFI total score	58.92 (17.36)	–	–	–
*MFI subscales*				
Physical fatigue	15.17 (4.21)	–	–	–
Mental fatigue	7.45 (4.42)	–	–	–
General fatigue	13.45 (4.53)	–	–	–
Reduced activity	12.42 (5.02)	–	–	–
Reduced motivation	10.42 (4.13)	–	–	–

ALS, amyotrophic lateral sclerosis; HC, healthy controls; ALSFRS‐R, ALS Functional Rating Scale—Revised; NIV, noninvasive ventilation; PEG, percutaneous endoscopic gastrostomy; ECAS, Edinburgh Cognitive and Behavioral ALS Screen; DAS, Dimensional Apathy Scale; STAI, State‐Trait Anxiety Inventory; MFI, Multidimensional Fatigue Inventory.

Of the overall patient sample, only 30 patients (80% men; mean age: 58.17 ± 10.15 years; mean education level: 10.77 ± 4.30 years; mean ALSFR‐R: 42.33 ± 3.18; mean disease duration: 20.06 ± 20.32 months) underwent functional RS‐fMRI scanning, and their clinical features did not differ from the remaining patients, except for ALSFRS‐R and male percentage, which were higher for fMRI‐scanned patients (data not shown). In this subset of ALS patients, the clinical measures, including fatigue assessment, were completed at the time of the scan. No patient had frontotemporal dementia. On the base of ECAS subscores (Poletti et al., 2016; Siciliano et al., 2017), according to the Strong criteria for frontotemporal spectrum disorder of ALS (Strong et al., 2017), within the whole group of ALS patients, 21 patients had cognitive impairment (ALSci) with executive dysfunction, and 11 patients had both executive and language impairments. Within the group of ALS patients who underwent MRI, 6 patients had ALSci with executive dysfunction and 3 patients had ALSci with both executive and language impairments. The mean total MFI score was 58.9 ± 16.3 in the whole population and 54.9 ± 17.9 in the subset of patients who underwent MRI, confirming that fatigue was a common symptom in the present series of patients with ALS.

Bivariate regression analyses showed that higher age, lower subscores on the subscale of memory of the ECAS, and higher scores at the executive subscale of the DAS were associated with higher scores on the “mental fatigue” subscale of the MFI. Moreover, lower “respiratory” subscores at the ALSFRS‐R and higher scores at the STAI form Y‐1 were related to higher scores on the “physical fatigue” subscale of MFI (Table 2). The variables statistically significant in univariate analyses were entered in two separate multivariate regression analyses as independent variables, with MFI‐physical fatigue as the dependent variables. This last analytic step identified age (at clinical observation) as an independent factor significantly associated with MFI‐mental fatigue and the “gross motor” subscore of ALSFRS‐R along with STAI form Y‐1 as independent factors significantly associated with MFI‐physical fatigue (Table 3).

**TABLE 2:** Bivariate regression analyses of Mental and Physical Fatigue subscales of Multidimensional Fatigue Inventory on demographic, clinical, motor, and extra‐motor variables

	Mental fatigue				Physical fatigue			
Variables	*B*	SE	β	*p*	*B*	SE	β	*p*
*Demographics*								
Age	0.12	0.04	0.26	**<.01**	0.07	0.04	0.15	.08
Education, years	–0.11	0.08	–0.11	.20	0.00	0.08	0.00	.96
Sex^a^	0.17	0.80	0.01	.83	1.25	0.75	0.14	.10
*Clinical and motor features*								
Disease duration, months	0.01	0.01	0.07	.39	0.01	0.01	0.08	.31
ALSFRS‐R total score	0.01	0.05	0.02	.77	–0.04	0.05	–0.07	.38
*ALSFRS‐R subscores*								
Bulbar	0.34	0.19	0.15	.08	0.11	0.19	0.05	.53
Fine motor	0.02	0.12	0.01	.85	–0.01	0.11	–0.01	.87
Gross motor	–0.09	0.12	–0.07	.42	–0.29	0.11	–0.22	**.01**
Respiratory	0.10	0.22	0.04	.62	–0.06	0.21	–0.02	.75
Upper Motor Neuron score	–0.13	0.09	–0.13	.14	0.00	0.08	0.00	.92
Patients under NIV^b^	–1.90	1.45	–0.11	.19	0.25	0.13	0.01	.85
Patients with PEG^b^	–0.81	2.60	–0.02	.75	2.90	2.46	0.10	.24
Patients in Riluzole therapy^b^	2.87	1.52	0.16	.06	0.01	1.46	0.00	.99
*Extra‐motor features*								
ECAS total score	–0.03	0.02	–0.12	.15	–0.03	0.02	–0.14	.10
*ECAS subscores*								
Executive functions	–0.03	0.04	–0.06	.49	–0.05	0.04	–0.11	.19
Fluency	–0.01	0.06	–0.02	.78	–0.10	0.06	–0.14	.09
Language	–0.08	0.08	–0.08	.33	–0.05	0.08	–0.05	.54
Memory	–0.20	0.08	–0.21	**.01**	–0.06	0.07	–0.07	.42
Visuospatial abilities	–0.36	0.27	–0.11	.19	–0.14	0.26	–0.04	.58
Beck Depression Inventory‐II	–0.02	0.04	–0.04	.60	0.08	0.04	0.17	.05
STAI form Y‐1	0.00	0.03	0.01	.88	0.07	0.03	0.21	**.01**
DAS total score	0.07	0.04	0.16	.06	0.00	0.04	–0.01	.85
*DAS subscales*:								
Executive	0.27	0.09	0.24	**<.01**	0.02	0.09	0.02	.76
Emotional	0.04	0.10	0.03	.67	–0.09	0.09	–0.08	.35
Cognitive and Behavioral Initiation	0.05	0.07	0.07	.41	0.01	0.06	0.01	.88
Epworth Sleepiness Scale	0.01	0.12	0.00	.92	0.00	0.11	0.00	.95

*Note*. Statistically significant variables are shown in bold.

^a^ Coded as: 0 = male, 1 = female.

^b^ Coded as: 0 = no, 1 = yes.

*B*, unstandardized beta coefficient; SE, standard error; β, standardized beta coefficient; ALSFRS‐R, ALS Functional Rating Scale—Revised; NIV, noninvasive ventilation; PEG, Percutaneous Endoscopic Gastrostomy; ECAS, Edinburgh Cognitive and Behavioral ALS Screen; DAS, Dimensional Apathy Scale; STAI, State‐Trait Anxiety Inventory; MFI, Multidimensional Fatigue Inventory.

**TABLE 3:** Multivariate regression analyses of Mental and Physical Fatigue subscales of Multidimensional Fatigue Inventory on demographic, clinical, motor, and extra‐motor variables resulting as statistically significant in bivariate analyses

		Coefficients			Multicollinearity			Model		
	*B*	SE	β	*p*	Tolerance	VIF	*F* test	*p*	*R*	*R* ^2^
Mental fatigue							5.60	**<.01**	0.34	0.12
Constant	2.86	2.73	–	.29	*–*	*–*				
Age	0.09	0.04	0.20	**.01**	0.94	1.06				
ECAS—memory	–0.14	0.08	–0.15	.09	0.89	1.11				
DAS—executive	0.16	0.10	0.14	.12	0.85	1.17				
Physical fatigue							5.67	**<.01**	0.28	0.08
Constant	14.48	1.57	–	**<.01**	**–**	**–**				
ALSFRS‐R gross motor	–0.26	0.11	–0.19	**.02**	0.98	1.01				
STAI form Y‐1	0.06	0.03	0.18	**.03**	0.98	1.01				

*Note*. *p* Values below .05 are shown in bold.

ECAS, Edinburgh Cognitive and Behavioral ALS Screen; DAS, Dimensional Apathy Scale; ALSFRS‐R, ALS Functional Rating Scale—Revised; DAS, Dimensional Apathy Scale; STAI, State‐Trait Anxiety Inventory; *B*, unstandardized beta coefficient; SE, standard error; β, standardized beta coefficient; VIF, variance inflation factor.

### 3.2 Resting‐State Network (RSN) functional connectivity analysis

Among RSNs, the sensorimotor (SMN), the default mode (DMN), the bilateral frontoparietal (FPN), and the salience (SLN) networks, showed statistically significant regional group effects in their spatial distribution (voxel‐level *p* ≤ .001, cluster‐level *p* ≤ .05). Final RS‐fMRI analysis was performed on 26 ALS patients compared to 26 HC because of the exclusion of four subjects for motion artifacts.

**FIGURE 2:**
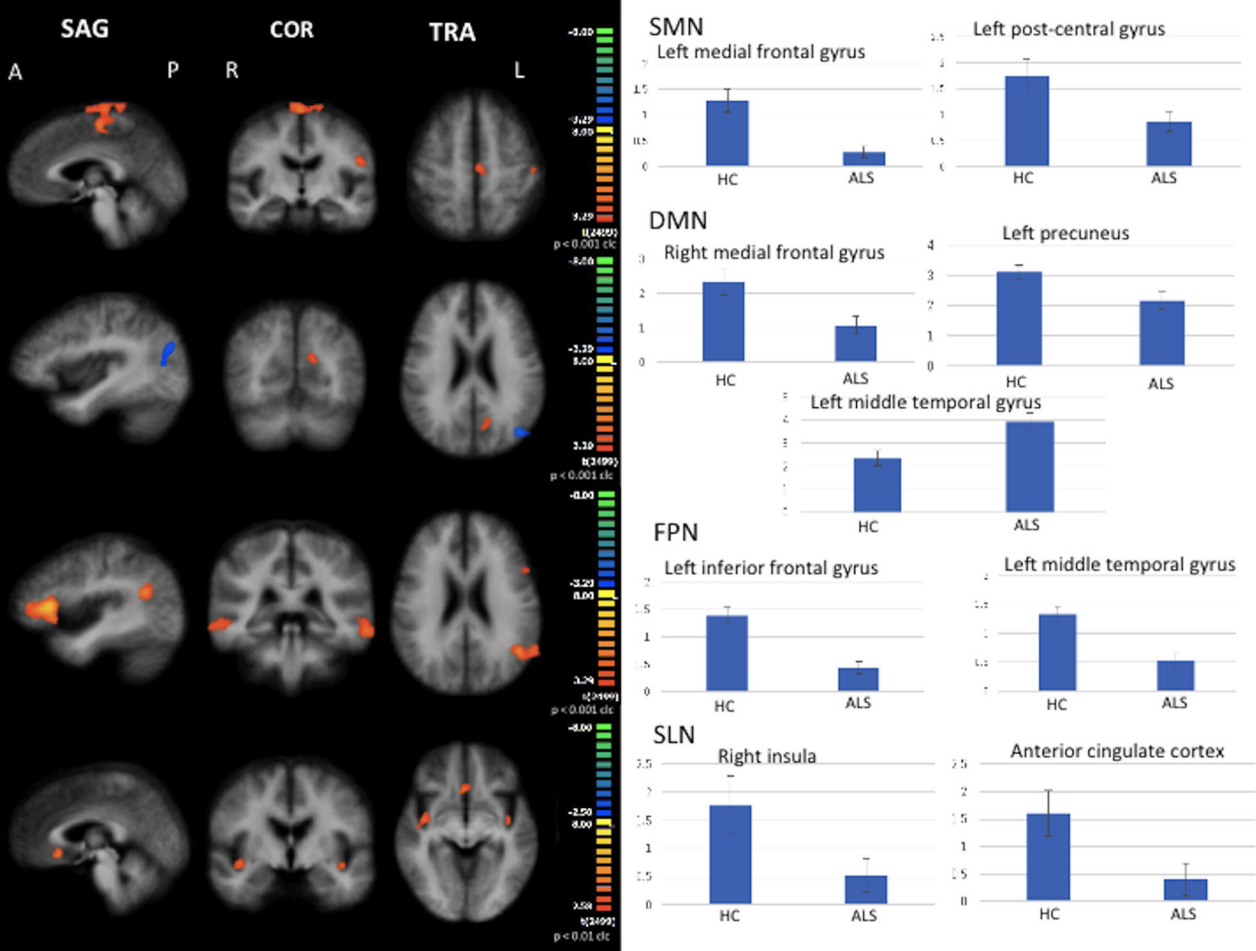
RSNs between‐group comparisons Functional connectivity (FC) signal fluctuations in ALS patients versus healthy controls (HC) in the sensorimotor network (SMN), in the default mode network (DMN), in the right and left frontoparietal networks (FPN), and in the salience network (SLN) (on the left, maps: right‐yellow scale for FC decrease, blue scale for FC increase; on the right, bar plots of the average FC levels). A = anterior; clc = cluster‐level corrected; COR = coronal; L = left; P = posterior; R = right; SAG = sagittal; TRA = transverse.

As for SMN, when compared to HC, ALS patients exhibited a decreased functional connectivity (FC) in the left medial frontal gyrus, and in the left postcentral gyrus (cluster‐level corrected *p* < .001) (Figure 2).

As for DMN, when compared to HC, ALS patients exhibited a decreased FC in the right medial frontal gyrus and left precuneus and an increased FC in the left middle temporal gyrus (cluster‐level corrected *p* < .001) (Figure 2).

As for FPN, when compared to HC, ALS patients exhibited a decreased FC in the right and left middle frontal gyrus and in the left inferior frontal gyrus (cluster‐level corrected *p* < .001) (Figure 2).

As for SLN, when compared to HC, ALS patients exhibited a decreased FC in the right and left anterior insular cortices and in the anterior cingulate cortex (cluster‐level corrected *p* < .01) (Figure 2).

Correlation analysis between mean *z* scores in the areas of abnormal FC in ALS patients and mean MFI subscores showed that the mean z‐score in the left middle frontal gyrus in the SMN and the subscore “reduced motivation” of MFI (*r* = 0.3986; *p* = .0437) were directly related. Additionally, the mean z‐score in the left medial temporal gyrus in the DMN and the subscore “mental fatigue” (MF) of MFI (*r* = −0.3953; *p* = .0457) were inversely related. Finally, the mean z‐score in the right insular cortex in the SLN and the subscore MF‐MFI (*r* = 0.3916; *p* = .0479) as well as the global MFI score (*r* = 0.3934; *p* = .0468), and the z‐score in the left insular cortex in the SLN and the subscore MF‐MFI (*r* = 0.408; *p* = .0385) were directly related.

## 4 DISCUSSION

This neuropsychological and neuroimaging study explored the cognitive and behavioral correlates of the dichotomic construct of fatigue in a large population of ALS patients and the RS‐fMRI correlates of the different components of fatigue in a subset of these patients who underwent MRI. Our findings revealed that a higher level of “mental fatigue” was significantly related to age and to some cognitive and behavioral performances of ALS patients (i.e., memory performance, as assessed by ECAS, and executive apathy, as assessed by DAS). Conversely, a higher level of “physical fatigue” was significantly associated with respiratory dysfunction and “gross motor” disability scores at ALSFRS‐R and higher levels of anxiety assessed by STAI form Y‐1. The analysis of the RS‐fMRI dataset, collected in a subset of ALS patients compared to age‐ and sex‐matched HC, revealed that mean FC *z* scores in several frontotemporal areas, showing impaired connectivity within DMN and SLN in the ALS group compared to HC, were significantly related to the mental component of fatigue in ALS patients.

### 4.1 Correlations of the physical component of fatigue with clinical scores in ALS

Some previous studies using neurophysiological measures, such as sustained isometric muscle contraction, to assess the severity of the physical component of fatigue in ALS patients, revealed predominant correlations of lower motor neuron impairment with fatigue (Vucic et al., 2007), although these measures have been shown to extend to more sites than those affected by clinical motor weakness (Sanjak et al., 2001). Additionally, further studies explored the correlations between measures of the severity of fatigue with some clinical features monitored in ALS (Chiò et al., 2021), such as motor functional status (Panitz et al., 2015), quality of life (An et al., 2022; Young et al., 2022), respiratory (Vogt et al., 2020), and sleep dysfunction (Lo Coco & La Bella, 2012). However, only a few scales evaluated more components of fatigue, such as the MFI and the checklist individual strength (CIS20‐R) (Vercoulen et al., 1994). Using this latter scale, a multidimensional scale capturing four dimensions of fatigue (i.e., subjective experience of fatigue, and reduction in concentration, motivation, and activity), Panitz et al. (2015) revealed a steady increase in physical, but also mental (motivation) fatigue during a 12‐month follow‐up in a cohort of ALS patients. Moreover, only the subscore of “subjective fatigue” (representing the reference for clinical levels of severity of fatigue) showed a correlation with the ALSFRS‐R score and the progression of the ALSFRS‐R after 12 months (Panitz et al., 2015). Other longitudinal studies found that fatigue severity increases during the disease course and was inversely related to age at disease onset (i.e., greater fatigue in youngest patients (Ramirez et al., 2008)), resulting in an independent predictor of depression (McElhiney et al., 2009). Although characterized by a cross‐sectional design, our correlation analysis confirmed the previously described association between a higher level of fatigue (especially in its physical component) and a greater “gross motor” and respiratory disability (as assessed by “respiratory” and “gross motor” subscores of ALSFRS‐R) (An et al., 2022; Panitz et al., 2015; Young et al., 2022) and higher levels of anxiety (Young et al., 2022). Young et al. (2022) clarified that anxiety and fatigue may represent the most common clinically indirect effects of dyspnea which play a relevant role in conditioning depression and quality of life in ALS.

### 4.2 Correlations of the mental component of fatigue with RS‐fMRI findings and behavioral and cognitive performances in ALS

In our population, age at clinical observation, together with impairment of memory performance and a greater (executive) apathy, was found directly correlated with a higher level of “mental fatigue,” suggesting that in ALS memory and behavioral dysfunction, as well as physiological age, may be related to the impairment of several brain areas inserted in cognitive networks underlying mental component of fatigue. Several studies revealed that, in health, the conditions of prolonged cognitively demanding tasks may induce cognitive fatigue in both young and older subjects (Hopstaken et al., 2016; Wang et al., 2016). From the neurophysiological point of view, Babu Henry Samuel et al. (2019) revealed that in a cohort of young subjects, as cognitive fatigue develops, behavior dysfunction and region‐ and time‐specific increase in neural EEG activity may be observed, suggesting a neural compensation. Differently, Babu Henry Samuel et al. (2019) found that behavioral performance did not decline in the older cohort as the experiment progressed and did not show neural compensation, suggesting less capacity to recruit additional resources in response to mental fatigue in older age. Probably, as revealed in our cohort of patients, mental fatigue is present at baseline in ALS, also in absence of prolonged cognitive performances, and this fatigability at rest may reflect the deleterious effects of the neurodegenerative process, widely demonstrated to be extended to both motor and extra‐motor areas by structural and functional neuroimaging studies (Agosta et al., 2013; Castelnovo et al., 2020; McKenna et al., 2021). In this regard, the results of our correlation analysis underlined the central origin of mental fatigue in ALS, revealing that decreased FC in the left middle temporal gyrus (DMN) was related to increased mental fatigue in ALS patients and decreased FC in the right and left insular cortices (SLN) were related to decreased mental fatigue in ALS patients. Similar functional changes have been revealed in insular areas associated with SLN in a cohort of patients with Parkinson's disease (PD), showing an inverse correlation of metabolic changes, displayed by using 2‐[18F] fluoro‐2‐deoxy‐Dglucose positron emission tomography, with the severity of fatigue (Cho et al., 2017). Conversely, this cohort of PD patients had a predominant impairment of metabolic activity in posterior areas of the DMN (Cho et al., 2017), differently from the pattern of FC impairment in anterior areas of DMN observed in our cohort of ALS patients. Remarkably, the preferential impairment of FC in the anterior areas of the DMN in ALS, as reported by numerous RS‐fMRI studies (Agosta et al., 2013; Tedeschi et al., 2012), recalls similar overlapping RS‐fMRI abnormalities observed in the behavioral variant of frontotemporal dementia (Trojsi et al., 2015) thus corroborating the evidence of a clinic‐radiological continuum between the two diseases. Moreover, more recent longitudinal RS‐fMRI evidence corroborated the theory that abnormalities of brain networks progressed over time within the frontostriatal and the frontoparietal networks in ALS patients and are related to frontal‐executive dysfunction (Castelnovo et al., 2020).

### 4.3 Limits

There are some limitations to our study. First, the patient sample that underwent MRI was relatively small. Second, longitudinal neuropsychological data was not available in the studied patients, hindering monitoring of fatigue and the cognitive and behavioral profile over time. Third, no fatigue assessment and neuropsychological scores, except for MMSE, were available in the HC group.

## 5 CONCLUSIONS

The two components of fatigue have different clinical correlates in ALS, showing a significant correlation between the physical component of fatigue and anxiety and respiratory/gross motor dysfunction, and between the mental component of fatigue and memory and apathy. Moreover, the mental component of fatigue is confirmed to have a putative central origin as implied by the significant correlation between MF‐MFI score and FC changes in some extra‐motor areas of the DMN and the SLN. These findings may give further clues for clarifying the dichotomic nature of fatigue in ALS and for a suggesting a better management of this symptom, including, for example, the possibility of using brain stimulation techniques in future longitudinal studies aimed at monitoring fatigue components across disease progression in larger cohorts of ALS patients.

## FUNDING

No funding was received for this research.

## CONFLICT OF INTEREST STATEMENT

The authors declare that this research was conducted in the absence of any commercial or financial relationships that could be construed as a potential conflict of interest.

### ETHICS STATEMENT

The research was conducted according to the principles expressed in the Declaration of Helsinki. Ethics approval was obtained from the Ethics Committee of the University of Campania “Luigi Vanvitelli” in Naples, Italy (Prot. n. 591/2018).

### PATIENT CONSENT STATEMENT

Patient/HC or family has written informed consent was obtained from each participant.

### PEER REVIEW

The peer review history for this article is available at https://publons.com/publon/10.1002/brb3.2931.

## Data Availability

All data and materials support the reported claims and comply with standards of data transparency. Deidentified data will be shared on reasonable request with the corresponding author.
